# Phytotoxicity of coal waste elutriates (Douro Coalfield, North Portugal) in *Lactuca sativa*

**DOI:** 10.1007/s11356-023-29868-w

**Published:** 2023-09-22

**Authors:** Bárbara S. Diogo, Aracelis Narayan, Catarina Mansilha, Jorge Espinha Marques, Deolinda Flores, Sara C. Antunes

**Affiliations:** 1 Instituto de Ciências Biomédicas de Abel Salazar (ICBAS), Universidade do Porto, Rua de Jorge Viterbo Ferreira, 228, 4050-313 Porto, Portugal; 2 Centro Interdisciplinar de Investigação Marinha e Ambiental (CIIMAR), Universidade do Porto, Terminal de Cruzeiros do Porto de Leixões, Avenida General Norton de Matos S/N, 4450-208 Matosinhos, Portugal; 3 Departamento de Biologia da Faculdade de Ciências da Universidade do Porto (FCUP), Rua do Campo Alegre s/n, 4169-007 Porto, Portugal; 4 Instituto de Ciências da Terra (ICT), Universidade do Porto, Polo Porto, 4169-007 Porto, Portugal; 5 Departamento de Geociências, Ambiente e Ordenamento do Território, Faculdade de Ciências da Universidade do Porto (FCUP), Rua do Campo Alegre, 4169-007 Porto, Portugal; 6 Departamento da Saúde Ambiental, Instituto Nacional de Saúde Doutor Ricardo Jorge, 4000-055 Porto, Portugal; 7 LAQV/REQUIMTE, Universidade do Porto, 4050-083 Porto, Portugal

**Keywords:** Coal mining, Self-burning, Elutriates, Ecotoxicology, Germination assay, *Lettuce*

## Abstract

One of the most important mining areas in the Douro Carboniferous Basin is the Pejão Coalfield. In the summer of 2017, a wildfire promoted the ignition and self-burning some of the coal waste piles in the area and caused important environmental changes, promoting a new heterogenic pedological zonation. This study aims to assess the ecotoxicological effects of 25 soil elutriates from these different soil types in seed germination and individual (emergence, growth, and morphologic alterations) and subindividual parameters in *Lactuca sativa*. The different evaluated endpoints were differently affected regarding the soil elutriate revealing the high heterogeneity of soil characteristics. The presence of different potentially toxic elements (e.g., Cd, Cr, Pb, Zn) in soil elutriates, even in low concentrations, caused effects on *L. sativa* development. Unburned coal wastes and downhill soil elutriates were able to inhibit the germination of *L. sativa* and affect them individually and sub-individually (decrease in size, biomass, and presence of morphological alterations). Additionally, it was observed that all soil elutriates induce a decrease in root size. The results highlight the importance of using elutriate samples in phytotoxicity studies of coal mining waste, since the tailings lixiviate may reduce plant establishment and growth, affecting the terrestrial ecosystems. The integrated use of seed germination assays with the analysis of morphological and biochemical alterations in plants proved to be sensitive parameters to evaluate the phytotoxicity of coal mining wastes.

## Introduction

Coal has been the main energy source over the last two centuries, playing a vital role in global energy needs and being critical to promote socioeconomic development (WCA 2022). Despite these advantages, coal mining waste (CMW), generated by coal extraction, is a serious environmental issue (Wang et al. 2020), and the proper management of CMW has become an important challenge (Liu et al. 2018). The environmental degradation caused by waste disposal is considered a major concern by several governmental agencies since these materials are subjected to intense geochemical and mineralogical processes (Espinha Marques et al. 2021) which can increase the dispersion of organic and inorganic pollutants (Ribeiro et al. 2014), as well as the leaching of potentially toxic elements (PTE) to the different matrices (e.g., soil, groundwater, freshwater) (Galhardi and Bonotto 2016; Espinha Marques et al. 2021). Furthermore, the possibility of bioaccumulation and biomagnification in the food web can also represent a high risk to the environment and wildlife (Stefaniak and Twardowska 2010).

The Douro Carboniferous Basin is one of the most important coal deposits in the North of Portugal, where anthracite A was mined in the São Pedro da Cova and Pejão Coalfields and whose activity resulted in more than 20 coal waste piles, located in the areas surrounding the old mines. These coal waste piles have been characterized (geochemical, mineralogical, and petrographic studies) in the last several years (e.g., Ribeiro et al. 2010a, b, 2014, 2020, 2022); however, without proper biological characterization and environmental control [e.g., Ribeiro et al. (2010a, 2014, 2020, 2022)]. One of the main problems of these waste piles is the potential for self-burning and the potential ignition due to wildfires (Ribeiro et al. 2010a, 2022). The burned and unburned CMW areas can affect the terrestrial ecosystems, changing the vegetation, and reducing plant establishment and growth (Hall et al. 1982; Stefaniak and Twardowska 2010). On the other hand, the combustion process causes changes in trace elements (e.g., Fe, Cu, Mn, and Ni), with some becoming more easily mobilized to surrounding soils and water bodies by percolation, lixiviation, or deposition of solid particles (Hall et al. 1982; Ahmad et al. 2009; Calabró et al. 2022; Zerizghi et al. 2022). Several studies regarding coal mines and CMW focus on the chemical analyses of the polluted soils but neglect the assessment of CMW leachates and the bioavailability of their contaminants for the water ecosystem (e.g., Agnieszka et al. 2014). Indeed, this approach is important to assess the water and sediment contamination level, but it is insufficient to evaluate the ecotoxicological effects on different species (Geffard et al. 2007).

*Lactuca sativa* is a model plant species, recommended by several international organizations for assessing the ecological effects of toxic substances (ISO 1995; USEPA 1996; OECD 2006). Phytotoxicity assays using *L. sativa* are simple, quick, reliable, inexpensive, and do not require expensive equipment (Park et al. 2016; Lyu et al. 2018). In particular, the seed germination and root elongation tests are some of the simplest methods to assess phytotoxicity since seed germination is the first exchange interface between the developing plant and the environment (W.H.O 1998). Several studies reported the sensitivity of *L. sativa* to several contaminants, metals, phenolic compounds, and effluents with the inhibition of root elongation (Lyu et al. 2018; Calabró et al. 2022). Indeed, Park et al. (2016) and Lyu et al. (2018) showed inhibition of 50% of root elongation after *L. sativa* exposure to fluorine and Zn. Mtisi and Gwenzi (2019) investigated the effect of coal ash application on metal bioavailability and uptake, and the results showed that coal ash did not affect the germination indices, but reduced lettuce growth and edible biomass yield. *L. sativa* has shown a relationship with the bioavailability and bioaccumulation of Zn, Cu, and Pb, and there is a relation between toxicological endpoints correlated with metal uptake and mining waste concentration (Calabró et al. 2022). Overall, the evaluation of contaminant impacts in terrestrial species is conducted in soil samples; however, it is important to study the environmental hazard posed by contaminants available in the soil aqueous phase, for terrestrial and aquatic plants, through soil elutriate assays (Loureiro et al. 2005; Antunes et al. 2010). Several authors have already described that soil elutriate assays are sensitive tools to assess soil retention capacity and the potential effects of the bioavailable fraction of contaminants present in soils/sediments of mining waste (Loureiro et al. 2005; Sackey et al. 2020).

The objective of this study was to assess the ecotoxicological effects in *Lactuca sativa* of elutriates from Technosols from a coal mine waste pile. A seed germination bioassay was conducted, and individual (emergence, growth, and morphologic alterations) and sub-individual parameters [catalase (CAT) activity, malondialdehyde (MDA), and total chlorophyll contents] were evaluated in the exposure individuals.

## Material and methods

### Study site

**Fig. 1:**
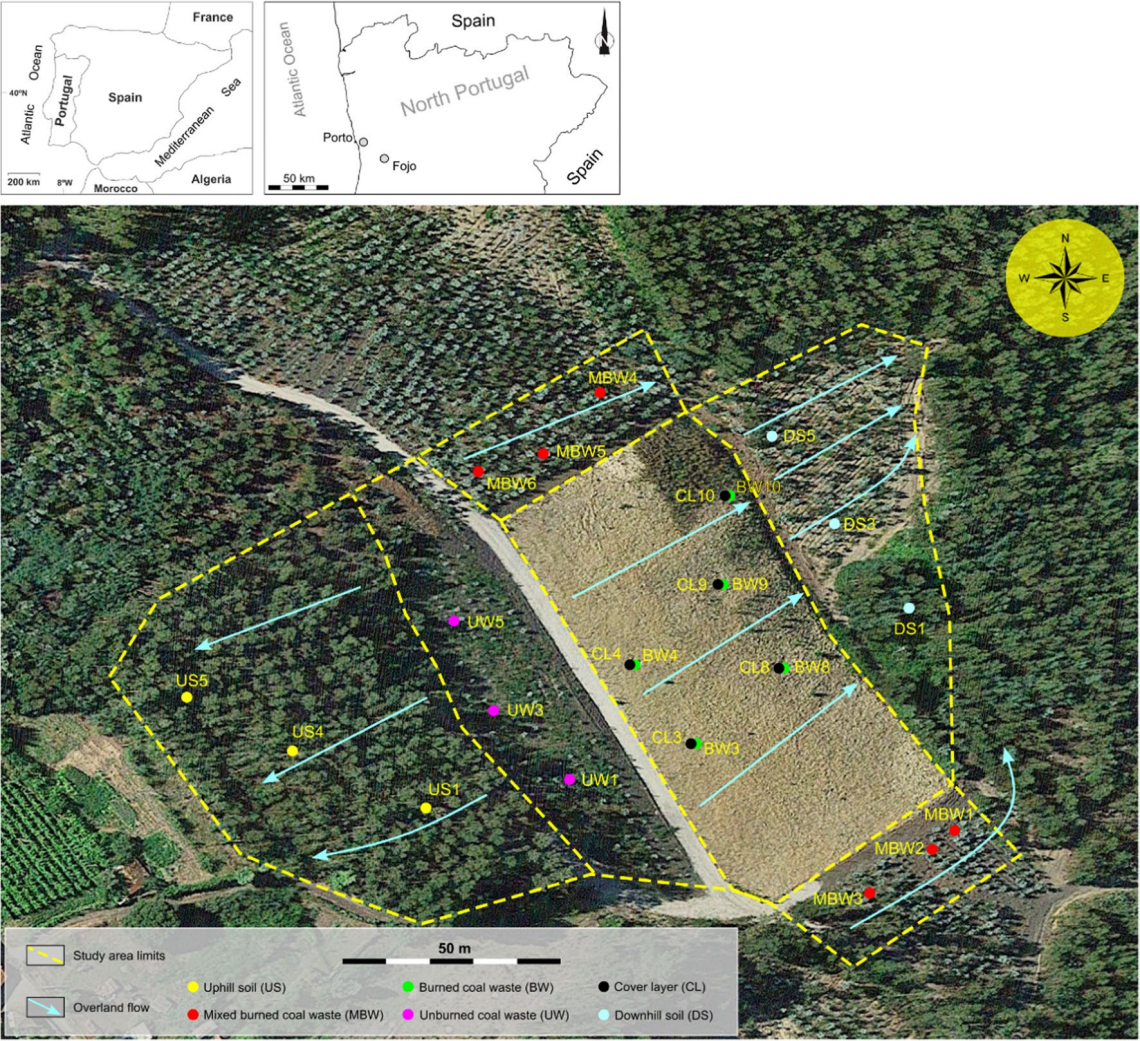
Fojo mine coal waste disposal area and pedological zonation, with the location of the samples sites

This study was carried out in the Fojo coal mine waste pile, located in the Pejão Coalfield (Douro Carboniferous Basin, North Portugal). As a result of coal mining between 1920 and 1994, several waste piles were geographically dispersed (Caetano 1998; Costa et al. 2022). In October 2017, a wildfire caused the ignition and self-burning of coal waste, and between 2017 and 2019, an effort to extinct the fire was done by remobilizing the coal-waste material, using water mixed with a cooling accelerator agent. The fire as well as the intervention for its extinction originated important environmental changes in the waste pile area and its surroundings and promoted a new pedological zonation (Fig. 1) characterized by: (i) uphill soil (US) — soil without mining influence, with Regosol/Cambisol features; (ii) unburned coal waste (UW) — without self-burning, with Technosol features; (iii) burned coal waste (BW) — with self-burning coal waste material, with Technosol features; (iv) protective cover of the burned coal waste (CL) — 30- to 40-cm-thick cover layer made of geological material from another site deposited after the extinction of the fire, with Technosol features; (v) mixed burned coal waste (MBW) —which contained both burnt and unburnt mixed material as a result of the remobilization process of the waste material during the extinguishing the fire and subsequently tilled for eucalyptus plantation, with Technosol features; (vi) downhill soil (DW) — situated downhill from the BW material, with Regosol/Cambisol/Anthrosol features.

### Sampling and general soil physical and chemical characterization

Soil samples (20 cm of depth from A horizon for US and DS, 20 cm of superficial layer for UW, MBW, for CL, and the upper 20 cm for BW) from 25 sites of the heterogenic area (see description in “Study site” — Fig. 1) were collected and stored in opaque containers. At the laboratory, the samples were stored at 4 °C, until beginning the assays. Soil pH and electrical conductivity were measured on the sampling day, in a soil-water suspension (extraction ratio 1:5 w/v) according to the method described in FAOUN (1984). Organic matter (OM) content and water holding capacity (WHC) of each soil were also measured according to SPAC (2000) and ISO (2008), respectively [for more details see Antunes et al. (2008)]. The bioavailable heavy metals (Cr, Mn, Ni, Cu, Zn, As, Cd, and Pb), inorganic ions (Na^+^, K^+^, Mg^2+^, Ca^2+^, Li^+^, Cl, NO_3_^−^, F^−^, and SO_4_^2+^), and other components (Al, Fe, NO_2_^−^, NH_4_^+^, and SiO_2_) in elutriates were quantified according to Espinha Marques et al. (2021).

### *Lactuca sativa* — germination assay

The germination assay was conducted according to OECD guidelines (2006) adapted for a sterile square Petri dish, in a Hoagland’s/Agar solution with each soil elutriate (see below). To evaluate soil toxicity, elutriates (indirect way) were made by combining 1:4 (w/v) of each soil with distilled water (USEPA 1998). At room temperature, the suspension was mechanically stirred for 12 h, followed by a 12h period of deposition. The elutriates were obtained by decantation after the rest period and used immediately. Seeds of *Lactuca sativa* (Vilmorin, França) were sterilized in a bath of 5% sodium hypochlorite (NaClO) for 5 min, followed by 3 washes in sterile deionized water. After that, in a flow chamber, 10 seeds were placed in a sterile square Petri dish containing solid Hoagland’s solution (Hoagland and Arnon 1950) with 1.5% agar and 10 mL of each soil elutriate. Four square Petri dishes were prepared per soil elutriate (4 biological replicates). On the control plates, 10 mL of sterile deionized water was added to the Petri dish with solid Hoagland’s solution. The plates were placed vertically, under controlled conditions of temperature (20 ± 2 °C), photoperiod (16 h^L^:8 h^D^), and luminosity (~ 6000 lux).

**Fig. 2:**
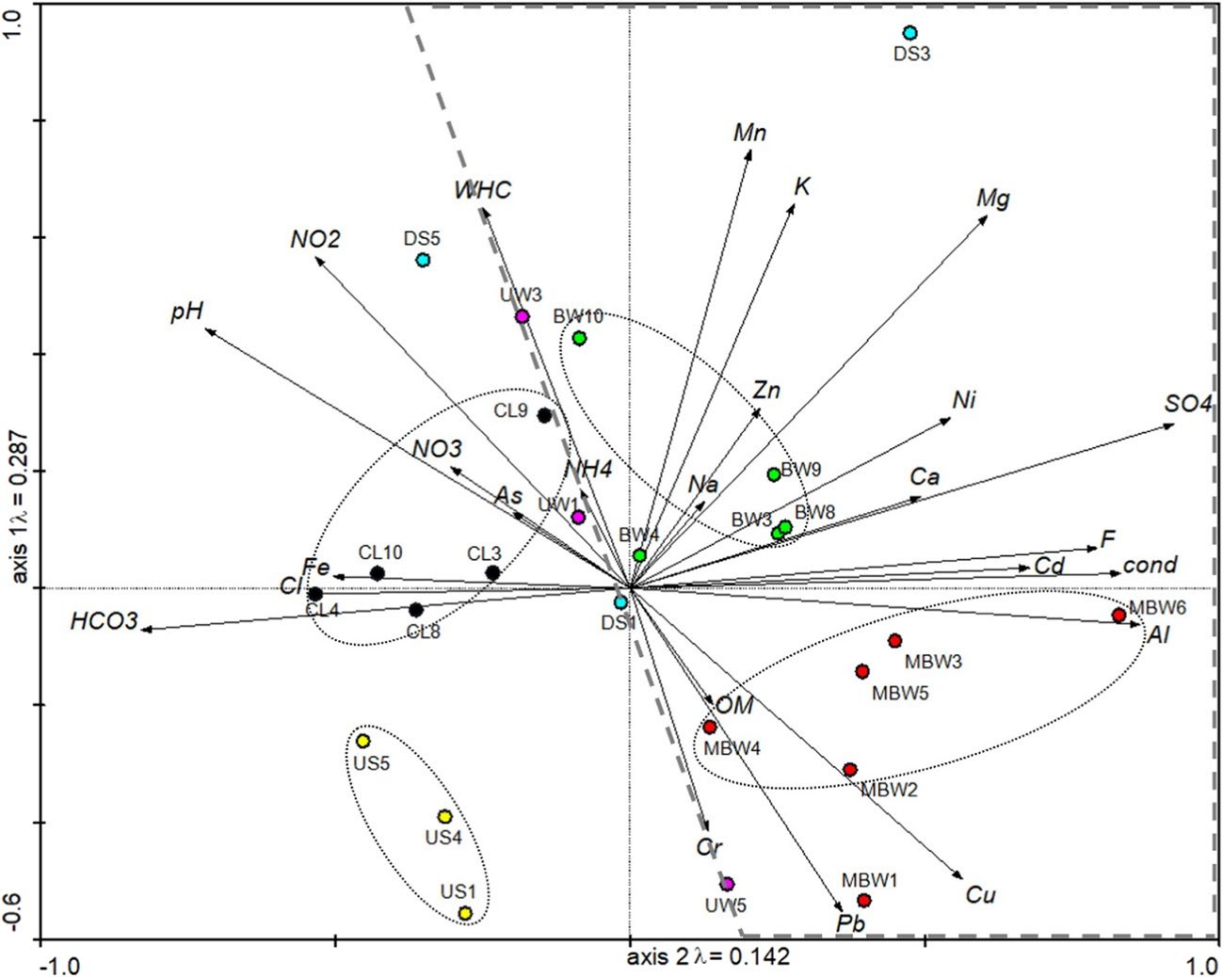
PCA result of physical and chemical parameters (abbreviations *see* “Sampling and general soil physical and chemical characterization”) measured in soil elutriates. Soil sampling areas are evidenced by oval dotted lines, and burned areas are all included inside the dashed line

At the end of the exposure period (14 days after 50% of seeds emergence in the control group), the percentage of seed emergence ([total number of germinated seeds/total number of initial seeds] * 100) and the biometric parameters of the seedlings [measurement of aerial part and roots size (cm), and fresh biomass (g) (OECD 2006)] were evaluated. Additionally, morphologic alterations in early plant development were recorded (e.g., damage/deformations, chlorosis, wilting; Fig. 2). For sub-individual effect evaluation, aerial parts were weighed and stored at − 80 °C for further quantification of total chlorophyll, and biochemical determinations of CAT activity and MDA content.

The total chlorophyll (> 5 mg of aerial part, *n* = 3) content was extracted in 1 mL of 96% ethanol at 4 °C overnight and determined according to Lichtenthaler (1987). After the extraction period, the absorbance at 644.2 and 648.6 nm was determined using a spectrophotometer (GenesysTM 10Series Thermo Spectronic). Results were expressed in mg/mg fresh weight.

For the determinations of CAT activity, samples (> 30 mg of aerial part per replicate, *n* = 3) were sonicated in 1 mL of ice-cold phosphate buffer (50 mM, pH 7.0) with 0.1% Triton X-100. Catalase is an antioxidant defense enzyme that decomposes hydrogen peroxide (H_2_O_2_) into water and molecular oxygen (Alkimin et al. 2020). CAT was quantified according to Aebi (1984), and the results were expressed as micromoles of H_2_O_2_ consumed/minute/mg fresh weight.

Regarding MDA quantifications, the samples (> 10 mg of aerial part, *n* = 3) were sonicated in 500 μL of 0.1 % trichloroacetic acid. The thiobarbituric acid method described by Elkahoui et al. (2005) was used to determine MDA content, and the results were expressed as μM MDA equivalents/mg fresh weight.

### Data analysis

A Principal Component Analysis (PCA) was performed to relate soil physical and chemical properties with the composition of soil elutriates. All the evaluated endpoints (germination inhibition, fresh biomass, size, and biochemical quantifications) were checked for normality by the Shapiro-Wilk test and for homogeneity of variances by Levene’s test. To determine significant differences between soil elutriates and the control elutriates, an analysis of variance (one-way ANOVA), followed by a Dunnett’s test, was performed to discriminate differences between the control group. The software SPSS Statistics v29 was used for all the statistical analyses, considering a significance level of *α* = 0.05.

## Results and discussion

A summary of the physical and chemical results of the soil elutriates is shown in Fig. 2. The Principal Component Analysis (PCA) demonstrates that the different soil samples from each pedological zone are true replicates since the spatial dispersion is similar. Additionally, PCA allowed the separation of soils according to their characteristics and origin. Mixed burned coal waste (MBW) and burned coal waste (BW) are associated with high values of heavy metals (e.g., Pb, Cu, Al, Cd, Ni, and Zn), and some inorganic ions (e.g., Na, K, Mg, and Ca), while unburned coal waste (UW) and the cover layer (CL) were characterized by high values of pH, NO_3_^−^, NO_2_^−^, and NH_4_ values (Fig. 2). The environmental impact of coal waste depends on several factors, namely, the type, concentration, solubility, mobility, and potential release of chemical elements (Kalembkiewicz and Sitarz-Palczak 2015; Espinha Marques et al. 2021). Some chemical elements are more easily leached and accessible for percolation and mobilization into the environment, namely, Ca, Mg, Na, Mn, Cd, Co, Ni, and Zn (Ribeiro and Flores 2021), and all these elements were quantified in high concentrations in the here-presented soil elutriates. Previous studies in coal mine waste piles (CMW) from the Douro Carboniferous Basin reported different concentrations of leachable elements found in self-burning coal waste samples *vs* unburned material since the leaching of elements can be temperature-dependent (Espinha Marques et al. 2021). Querol et al. (2008) reported the leaching potential of unburned and burned coal waste piles and concluded that the burned coal waste produces leachates with higher concentrations of Al, Ca, K, Mg, Mn, and Ni, which corroborates the results presented here. DS1, DS3, and DS5 soils (downhill soils with low vegetation diversity; *Eucalyptus* sp. and *Acacia* sp.), which are located downhill from the burned material (Fig. 1) with high leaching of PTE, appear not clustered, but rather dispersed (Fig. 2), possibly due to their heterogeneous pedological features. The opposite was observed in the uphill soils (US1, US4, and US5), which appeared in an isolated group, without association with the here-measured physical and chemical parameters (Fig. 2). This latter pedological zone corresponds to a forest that has not been exposed to the impact of coal mining (e.g., exploitation or waste deposit). Moreover, this area is considered an undisturbed zone with dense vegetation and high species diversity (e.g., bryophytes, pines, strawberry trees).

One of the most significant stages in a plant’s development is seed germination. This sensitive stage can be affected by several physical and chemical parameters (Bewley 1997; Seneviratne et al. 2019). Different studies already documented the effect of chemicals released from several industrial, agricultural, and mining effluents (such as PTE, pesticides, and fertilizers) in seed germination and seedling growth (Seneviratne et al. 2019; Alengebawy et al. 2021). The results from the germination assays regarding fresh biomass and size of *L. sativa* after exposure to soil elutriates are presented in Table 1. All the soil elutriates inhibited the seeds’ germination. However, only a set of 6 soils (UW3, UW5, MBW9, CL10, DS3, and DS5) caused a significant inhibition (> 30%) relative to the control group (*F*_[25, 111]_ = 3.252, *p* < 0.001). Despite the different physical and chemical characteristics of the soil elutriates (Fig. 2), it is important to emphasize that their chemical composition includes several elements that have already been shown to cause deleterious effects in plant growth, namely, Fe, Cr, Cu, Mn, and Zn. Seneviratne et al. (2019) considered that the inhibition of the seeds’ physiological and metabolic activities results in a decrease in germination, as a consequence of the presence of PTE (e.g., Cu, Zn, Cd, As) exposure. Some of these PTE act as micronutrients and participate in several metabolic reactions in plant development. However, when the threshold concentrations are exceeded, they become toxic (Angulo-Bejarano et al. 2021). Although the chemical elements detected in the soil elutriates appear in concentrations lower than those considered capable of affecting 50% of the individuals exposed (EC_50_) [e.g., the maximum [Ni] in the soil elutriates was 23.8 μg/L in CL3 and the EC_50_ (10 days) = 148 μg/L, (Lyu et al. 2018)], the fact is that these soil elutriates are complex samples and, possibly, more toxic than expected (Houshmandfar and Moraghebi 2011). Furthermore, Shafiq et al. (2010) showed that exposure to PTE mixtures decreases seed germination in plants, changes cell membrane permeability, and reduced seed reserves.

**Table 1:** Percentage of inhibition of germination (%), fresh biomass (g), and size (cm), observed in aerial part and roots of *Lactuca sativa* after exposure to soil elutriates. The percentage of morphologic alterations detected after exposure to soil elutriates (Fig. 4) is also presented. Significant differences are shown in bold (Dunnett test, *p* < 0.05)

Soil samples	Germination inhibition (%)	Fresh biomass (g)		Size (cm)		Morphological alterations (%)	
		Aerial part	Roots	Aerial part	Roots	Aerial part	Roots
CTL	1.7	0.39 ± 0.02	0.023 ± 0.003	5.22 ± 0.32	4.57 ± 0.04	0.0	0.0
US1	2.5	0.41 ± 0.03	0.018 ± 0.002	4.23 ± 0.14	**1.87 ± 0.30**	5.0	10.0
US4	27.5	0.31 ± 0.02	0.017 ± 0.002	3.93 ± 0.44	**1.38 ± 0.14**	**35.0**	**47.5**
US5	17.5	0.29 ± 0.03	0.025 ± 0.003	4.39 ± 0.39	**1.88 ± 0.19**	22.5	25.0
UW1	17.5	**0.21 ± 0.02**	0.012 ± 0.001	**3.27 ± 0.74**	**1.77 ± 0.48**	**35.0**	**50.0**
UW3	**45.0**	**0.08 ± 0.03**	**0.005 ± 0.001**	**2.52 ± 0.23**	**0.84 ± 0.15**	**65.0**	**65.0**
UW5	**37.5**	**0.19 ± 0.02**	**0.008 ± 0.001**	**3.63 ± 0.48**	**2.70 ± 0.35**	**42.5**	**45.0**
MBW1	2.5	0.29 ± 0.03	**0.010 ± 0.001**	5.30 ± 0.32	3.28 ± 0.41	2.5	7.5
MBW2	20.0	**0.19 ± 0.03**	**0.006 ± 0.002**	4.15 ± 0.49	**1.80 ± 0.26**	27.5	32.5
MBW3	5.0	0.29 ± 0.03	**0.007 ± 0.002**	5.59 ± 0.58	**2.14 ± 0.49**	7.5	10.0
MBW4	10.0	0.31 ± 0.03	0.014 ± 0.002	4.94 ± 0.35	**2.45 ± 0.56**	12.5	17.5
MBW5	15.0	**0.18 ± 0.03**	0.012 ± 0.003	**3.42 ± 0.65**	**1.34 ± 0.29**	22.5	**37.5**
MBW6	17.5	0.27 ± 0.02	0.017 ± 0.003	5.10 ± 0.47	**1.87 ± 0.16**	17.5	30.0
BW3	15.0	0.32 ± 0.03	0.034 ± 0.004	4.45 ± 0.23	**2.80 ± 0.75**	20.0	27.5
BW4	22.5	0.29 ± 0.03	0.033 ± 0.004	4.04 ± 0.59	**2.21 ± 0.54**	22.5	**42.5**
BW8	15.0	**0.14 ± 0.04**	**0.007 ± 0.003**	**2.33 ± 0.60**	**1.11 ± 0.26**	**42.5**	**62.5**
BW9	**35.0**	**0.17 ± 0.03**	**0.010 ± 0.003**	4.31 ± 0.18	**2.01 ± 0.26**	**35.0**	**47.5**
BW10	10.0	0.37 ± 0.02	0.027 ± 0.002	4.30 ± 0.53	**2.10 ± 0.33**	7.5	27.5
CL3	20.0	**0.22 ± 0.02**	0.021 ± 0.003	**3.49 ± 0.50**	**2.00 ± 0.46**	27.5	**42.5**
CL4	12.5	**0.28 ± 0.02**	0.027 ± 0.003	4.56 ± 0.30	**1.64 ± 0.43**	5.0	17.5
CL8	17.5	0.30 ± 0.02	0.027 ± 0.003	4.07 ± 0.33	**2.12 ± 0.31**	20.0	30.0
CL9	22.5	**0.22 ± 0.04**	0.012 ± 0.002	**3.53 ± 0.31**	**1.39 ± 0.23**	**30.0**	**42.5**
CL10	**40.0**	**0.20 ± 0.04**	0.020 ± 0.003	4.51 ± 0.43	**2.31 ± 0.25**	**47.5**	**45.0**
DS1	20.0	0.44 ± 0.04	**0.003 ± 0.003**	**3.33 ± 0.58**	**1.97 ± 0.28**	**32.5**	32.5
DS3	**30.0**	**0.16 ± 0.03**	0.024 ± 0.003	**2.96 ± 0.53**	**2.04 ± 0.40**	**40.0**	**45.0**
DS5	**37.5**	**0.20 ± 0.04**	0.012 ± 0.002	**3.00 ± 0.81**	**2.04 ± 0.50**	**50.0**	**65.0**

*L. sativa* exposure to soil elutriates UW3 and UW5 (unburned coal waste) caused also a significant decrease of fresh biomass and size in the aerial part (*F*_[25, 111]_ = 6.063, *p* < 0.001; *F*_[25, 111]_ = 4.037, *p* < 0.001, respectively) and roots (*F*_[25, 111]_ = 6.437, *p* < 0.001; *F*_[25, 111]_ = 6.822, *p* < 0.001, respectively) (Table 1). Despite not causing significant changes in seed germination, some soil elutriates were able to reduce plant height, root length, and fresh weight of the aerial part and roots of *L. sativa* (e.g., mixed burned coal waste: MBW2, MBW5; burned coal waste: BW8 and BW9; unburned coal waste: CL3 and CL9; and downhill soils: DS1, DS3, and DS5). Moreover, all the soil elutriates (except burned coal waste MBW1) caused a significant reduction in root length. Lyu et al. (2018) reported the success of root elongation tests with *L. sativa*, to assess the toxicity of phenolic compounds, pure chemicals, and complex effluent materials. Even though the mechanisms involved in the inhibition of root growth are not well established, as they are the first contact with the contaminated medium, they are generally more sensitive (Gangwar and Singh 2011). The inhibition of root growth may be related to the ability of PTE present in the samples to affect physiological, biochemical, and molecular processes (e.g., changes in nutrient flux and action, cell cycle interruption) (Geremias et al. 2012). Furthermore, the literature suggests that root growth inhibition (caused by PTE) alters water balance and nutrient absorption, affecting their transportation to the aerial parts and negatively affecting the shoot growth (Singh et al. 2016).

**Fig. 3:**
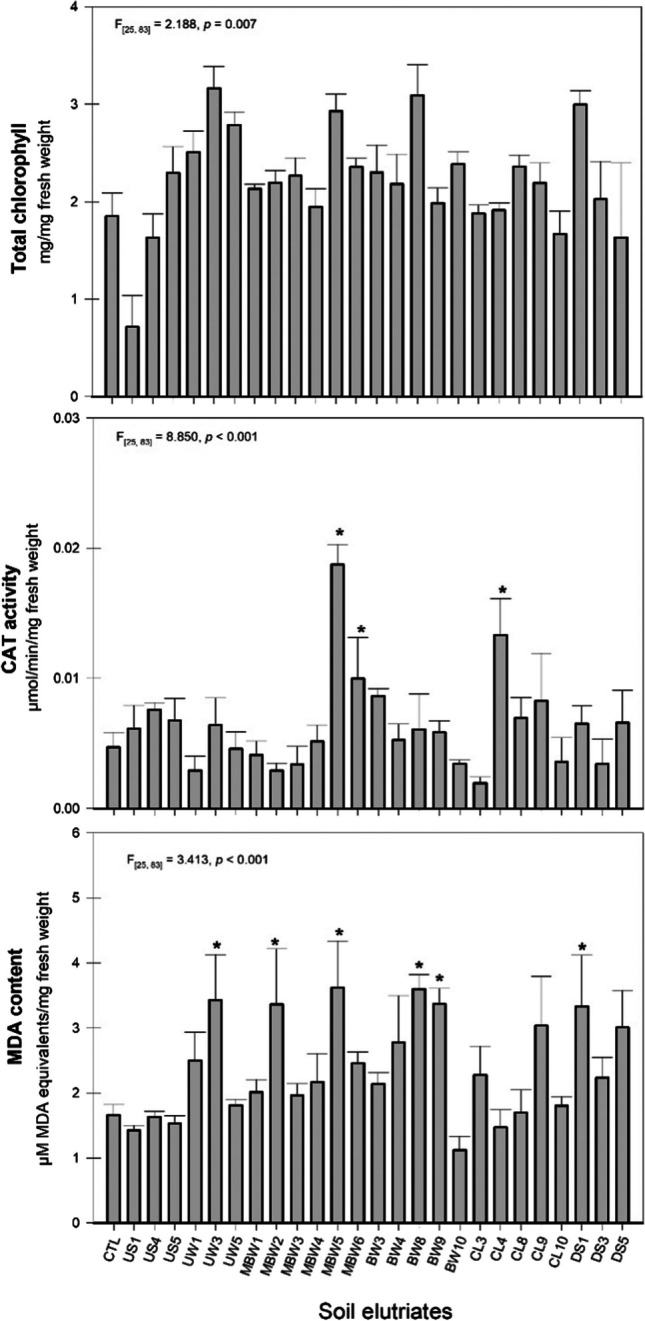
Results of *Lactuca sativa* pigments (total chlorophyll) and biochemical biomarkers [catalase (CAT) and malondialdehyde (MDA) measured in aerial part] after exposure to the different soil elutriates. Data are expressed as mean ± standard error; ANOVA results are presented at each figure’s top. *Significant differences when compared to CTL (Dunnett test, *p* < 0.05)

**Fig. 4:**
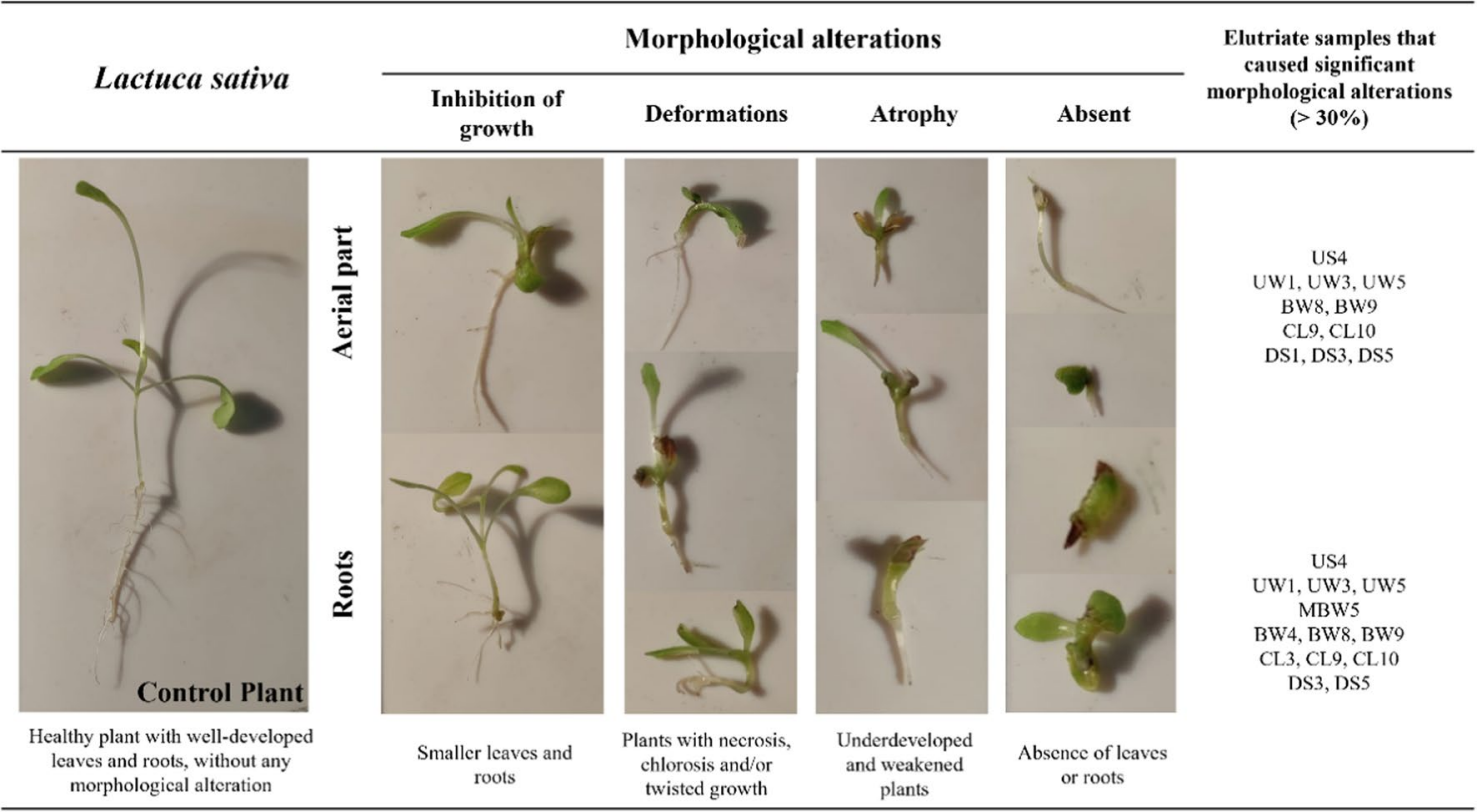
Description of morphological alterations observed in *Lactuca sativa* after exposure to different soil elutriates. The soil elutriates that induced significant morphological alterations (> 30 %, compared to the control group) in the aerial part and roots of *L. sativa* are also presented

In addition to the endpoints proposed by the guideline, the visual phytotoxicity assessment is also recommended (OECD 2006). Therefore, an evaluation of morphological, pigments content and biochemical alterations observed in the early plant development stage was recorded (Table 1; Figs. 3 and 4). Figure 4 exhibits and describes some alterations observed in the aerial part and roots after *L. sativa* exposure to soil elutriates (e.g., deformations, atrophy, reduced growth) as well as in the control plants. Severe toxicity symptoms (necrotic lesions, chlorosis, senescence, inhibition of growth) have already been reported in plants exposed to PTE (e.g., Cd, Cr, Pb, and Al) even at very low concentrations (mg/L) (Angulo-Bejarano et al. 2021). Singh et al. (2016) reported several symptoms observed in leaves, namely, chlorosis, senescence, low biomass accumulation, and inhibition of growth after heavy metals exposure (e.g., Cd, Zn, Fe, and Cu, in the order of mg/L). Moreover, soil elutriates that induced significant alterations, > 30 % of morphological alterations, were also identified in Table 1. A higher percentage of morphological alterations was observed in the aerial part (*F*_[25, 111]_ = 5.592, *p* < 0.001) and roots (*F*_[25, 111]_ = 5.452, *p* < 0.001) of *L. sativa* exposed to soil elutriates UW (unburned coal waste) and DS (downhill soils). The results demonstrated that these elutriates (with higher NH_4_^+^, NO_3_^−^, NO_2_^−^, and PTE concentrations, Fig. 2) inhibit *L. sativa* germination and growth (size and biomass, Table 1) and cause morphological phytotoxicity (Fig. 4; Table 1). Other studies have already described the toxicity symptoms of *L. sativa* exposed to PTE, inorganic ions, and other components detected in the soil elutriates (Singh et al. 2016; Angulo-Bejarano et al. 2021). Gangwar and Singh (2011) reported that typical symptoms of Cr toxicity involve slight leaf chlorosis and wilting and stunted growth of roots. Moreover, Gangwar and Singh (2011) showed that the accumulation of NH_4_ leads to nutrient deficiency and chlorosis in plants. Kohli et al. (2019) showed that Pb also affects the physiological characteristics of plants, reducing the seed germination rates and causing stunted growth in roots, necrotic lesions, and leaf chlorosis.

Although not considered in standardized protocols, other endpoints were also used to assess the toxicity of soil elutriates at the biochemical and sub-individual levels on seed germination and seedling development (e.g., total chlorophyll content, catalase activity, and malondialdehyde content). No significant alterations in total chlorophyll content were recorded relative to the control group (Fig. 3). Total chlorophyll content showed not be a responsive endpoint in the present study; however, it has already been considered a sensitive indicator of toxicity, being used as an indicator of the physiological stage of different species (e.g., Diogo et al. 2023; Gavina et al. 2013). Moreover, several authors reported changes in pigment content in different plants (e.g., *Phaseolus vulgaris*, *Zea mays*, *Lactuca sativa*) exposed to soil and soil elutriate samples with PTE (e.g., Cu, Ni, Pb, Zn, and Cd) (Pereira et al. 2009; Seneviratne et al. 2019; Alengebawy et al. 2021). These metals are considered destructive substances for photosynthesis, contributing to the declines, either causing structural damage to chloroplasts, preventing photosynthetic light harvesting, affecting the photosynthetic electron transport, or inhibiting chlorophyll biosynthesis (Sharma and Agrawal 2005; Sethy and Ghosh 2013; Seneviratne et al. 2019).

Regarding CAT activity, a significant increase was recorded in the aerial part after exposure to soil elutriates MBW5, MBW6, and CL4 (Fig. 3), suggesting that these soils might have caused a pro-oxidative perturbation in *L. sativa*. One of the typical defense mechanisms against toxic elements is the enhanced activity of antioxidant enzymes, which play an important role in the adaptation/survival of plants exposed to stress conditions (Zhang and Farahbakhsh 2007). CAT activity changes (inductions and inhibitions) in plants exposed to PTE (e.g., Cu, Cr, Pb, Zn) have already been shown to be directly related to metal concentrations. Gwozdz et al. (1997) reported that CAT activity decreases in the presence of high concentrations of PTE (e.g., Pb, Cb, and Cu) and increases in the presence of low concentrations, corroborating what was observed in the present study (in soil elutriates CL4, MBW5, and MBW6). According to the here-obtained results, CAT demonstrated to be able to neutralize reactive oxygen species (ROS) production and prevent oxidative damage possibly caused by MBW6 and CL4, since the MDA content was not affected by these samples. In contrast, the same did not happen for the MBW5 sample, which caused an increase in CAT activity, and a significant increase in MDA content (Fig. 3). MDA content was also increased after exposure to the soil elutriates of UW3, MBW2, BW8, BW9, and DS1 (Fig. 3) in the aerial part. The excessive formation of ROS and the consequent increase in MDA content in the aerial part, and roots, of plants exposed to different PTE (e.g., Cd, Cr, Pb, and Zn) was previously reported by several authors (Sethy and Ghosh 2013; Ding et al. 2021). MDA is a by-product of lipid peroxidation, used as an indicator of oxidative stress (Zhang and Farahbakhsh 2007). In this study, MDA demonstrated to be a sensitive parameter since it allowed identifying samples that can affect the metabolic and physiological pathways of *L. sativa*, despite no significant inhibition on seed germination occurring (Zhang and Farahbakhsh 2007).

## Conclusion

Although most studies focus only on the effect of coal mining wastes on plant germination and growth, evaluating the effect of elutriates from these contaminated soils is also a key subject, and this procedure should be a complementary tool in assessing the toxicity of these wastes. The different contaminants present in the wastes (e.g., PTE) are dispersed into the environment and reach the surrounding areas. Through a complementary approach with seed germination assays and individual (emergence, growth, and morphologic alterations) and sub-individual (biochemical biomarkers) parameters, the present study allowed to evaluate of the ecotoxicological effect on *L. sativa* of elutriates from soils with different features, according to the type of coal mining influence (soil without mining influence, located uphill from the coal waste, unburned coal waste, coal waste with self-burning and a protective cover, coal waste with mixed material and subsequently tilled for eucalyptus plantation, soil situated downhill from the covered and burned coal waste). The research results revealed that (i) despite being detected in lower concentrations than in soils, the bioavailable metals (e.g., Cr, Cd, Pb, Zn) present in the elutriates were able to affect *L. sativa* performance; (ii) all elutriates caused a decrease in root size (first contact surface with the elutriate); (iii) elutriates from unburned coal waste and downhill soil cause higher inhibition of germination and development of *L. sativa*, also causing morphological and biochemical alterations. The mobilization and percolation of bioavailable organic and inorganic pollutants (present in coal mining wastes) to the different surrounding matrices severely threaten the survival of plants. Consequently, they affect the biota of terrestrial and aquatic ecosystems and contribute to the disruption of ecological balance. This study demonstrated the relevance of testing the effect of elutriates from coal mining wastes to assess their toxicity and alert the society and scientific community to the impacts of contaminants dispersed (by percolation and mobilization) to adjacent areas and water ecosystems.

## Data Availability

All datasets generated or analyzed during this study are included in the manuscript.
